# Genetic manipulation and targeted protein degradation in mammalian systems: practical considerations, tips and tricks for discovery research

**DOI:** 10.1002/2211-5463.13581

**Published:** 2023-03-08

**Authors:** Stefano L. Giandomenico, Erin M. Schuman

**Affiliations:** ^1^ Max Planck Institute for Brain Research Frankfurt am Main Germany

**Keywords:** CRISPR, delivery, differentiation, neuroscience, TPD

## Abstract

Gaining a mechanistic understanding of the molecular pathways underpinning cellular and organismal physiology invariably relies on the perturbation of an experimental system to infer causality. This can be achieved either by genetic manipulation or by pharmacological treatment. Generally, the former approach is applicable to a wider range of targets, is more precise, and can address more nuanced functional aspects. Despite such apparent advantages, genetic manipulation (i.e., knock‐down, knock‐out, mutation, and tagging) in mammalian systems can be challenging due to problems with delivery, low rates of homologous recombination, and epigenetic silencing. The advent of CRISPR‐Cas9 in combination with the development of robust differentiation protocols that can efficiently generate a variety of different cell types *in vitro* has accelerated our ability to probe gene function in a more physiological setting. Often, the main obstacle in this path of enquiry is to achieve the desired genetic modification. In this short review, we will focus on gene perturbation in mammalian cells and how editing and differentiation of pluripotent stem cells can complement more traditional approaches. Additionally, we introduce novel targeted protein degradation approaches as an alternative to DNA/RNA‐based manipulation. Our aim is to present a broad overview of recent approaches and *in vitro* systems to study mammalian cell biology. Due to space limitations, we limit ourselves to providing the inexperienced reader with a conceptual framework on how to use these tools, and for more in‐depth information, we will provide specific references throughout.

## Graphical abstract

This article explores how to gain an understanding of the molecular pathways that control cell and organismal function through genetic manipulation. We discuss the use of different CRISPR‐Cas technologies, targeted protein degradation, and pluripotent stem cells to interrogate gene function. Our aim is to provide a broad and practical overview of available approaches and model systems to study mammalian biology.

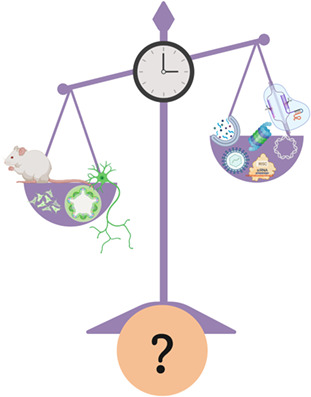

**Abbreviations**
AAV: adeno‐associated virus
AbTAC: antibody‐based PROTAC
AID: auxin‐inducible degron
aTAG: Achilles TAG
ATTEC: autophagosome‐targeting chimera
AUTAC: autophagy‐targeting chimera
CAKE: conditional activation of knock‐in expression
Cas: CRISPR‐associated (protein)
Cas9: CRISPR‐associated protein 9
Cas9n: Cas9 nickase
CBP: CREB‐binding protein
CDS: coding sequence
CRISPIE: CRISPR‐mediated insertion of exon
CRISPR: clustered regularly interspaced short palindromic repeats
CRISPRa: CRISPR activation
CRISPRi: CRISPR interference
dCas9: deactivated Cas9
DD‐Cas9: destabilizing domain Cas9
DMT3A: DNA (cytosine‐5)‐methyltransferase 3A
dTAG: degradation tag
EBNA1: Epstein–Barr nuclear antigen‐1
FACS: fluorescent activated cell sorting
FKBP12: FK506‐binding protein (FKBP)12
GFP: green fluorescent protein
GOF: gain‐of‐function
HDR: homology‐directed repair
HITI: homology independent targeted integration
HiUGE: homology‐independent universal genome engineering
hPSCs: human pluripotent stem cells
KRAB: Kruppel‐associated box
LOF: loss‐of‐function
LYTAC: lysosomal targeting chimera
M‐CREATE: multiplexed‐Cre‐recombination‐based AAV targeted evolution
mESCs: mouse embryonic stem cells
MMEJ: microhomology‐mediated end joining
NHEJ: nonhomologous end joining
ORANGE: open resource for the application of neuronal genome editing
ORF: open reading frame
p300: histone acetyltransferase p300
PAM: protospacer adjacent motif
POI: protein of interest
PROTAC: proteolysis targeting chimera
PSCs: pluripotent stem cells
RNAi: RNA interference
RNP: ribonucleoprotein (complex)
S/MAR: scaffold/matrix attachment region
sgRNA: single‐guide RNA
SID: SIN3‐interacting domain
ssODNs: single‐stranded oligo donors
SunTag: supernova tag
TALEN: transcription activator‐like effector nuclease
TKIT: targeted knock‐in with two (guides)
TPD: targeted protein degradation
UPS: ubiquitin‐proteasome system
WT: wild‐type

## Our question and how to best tackle it—models, manipulation, and read‐out

### Transient vs. stable modifications

**Fig. 1:**
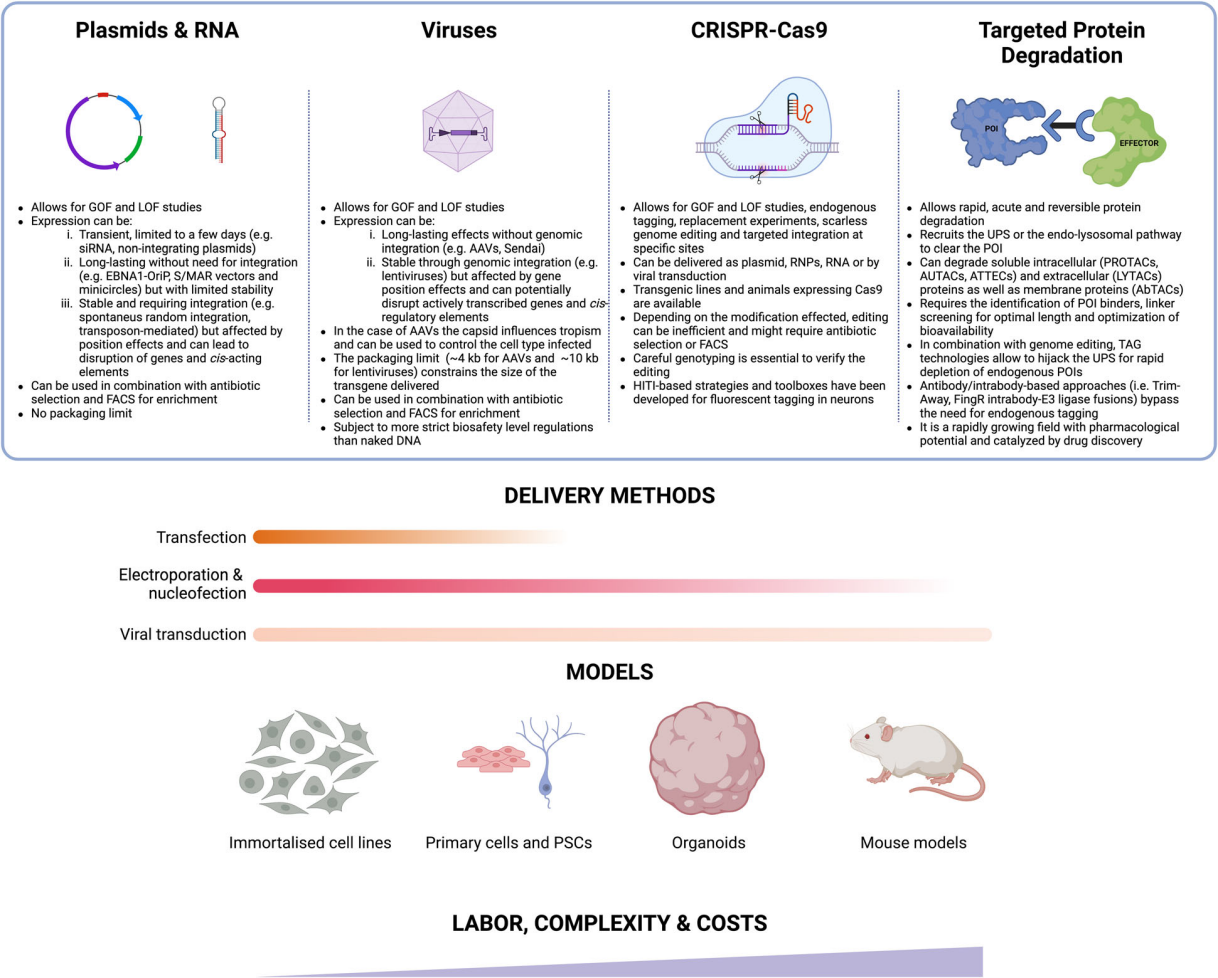
Genetic manipulation and TPD approaches, delivery methods, and model systems. The top table summarizes the different genetic manipulation approaches and TPD strategies available, and lists their advantages and limitations. The schematic below reports the different delivery methodologies and how well‐suited they are for the different model systems, the solid color indicates the method is well‐suited for the model system, and the fading color indicates decreased suitability. Broadly speaking, with increased complexity of the system, the associated costs and labor increase.

Molecular biology offers a number of tools and approaches for genetic manipulation and one should carefully consider their advantages and disadvantages before choosing a specific one. For example, if the experimental question under investigation can be addressed in an immortalized cell line that benefits from rapid growth and high transfection efficiency (e.g., HEK293, HeLa) then transient transfection of plasmids and siRNA/shRNAs might be sufficient to answer our questions [1]. However, this is not always possible. For example, it may be that the genes that make up the pathway of interest are not expressed in such cells. It could also be the case that the cell lines available cannot be efficiently transfected [2], or alternatively, the perturbation of the process investigated may require sustained construct expression, which exceeds the capabilities of a transient expression system [3] (Fig. 1).

Stable plasmid transfection and viral transduction with lentiviruses or AAVs, in combination with selection by fluorescence or antibiotic resistance, are straightforward approaches to overcome these limitations. In mitotically active cells, sustained transgene expression can be achieved either by integration of the expression cassette into the host genome or by delivery of an episomal vector that can replicate autonomously and get passed on to daughter cells through successive rounds of cell division [4]. Integration into the genome of cells can be achieved in a number of ways. For example, in certain transformed cell lines and in mouse embryonic stem cells (mESCs) delivery of a construct containing an antibiotic resistance cassette followed by selection is sufficient to achieve random genomic integration [5]. In our experience, the success rates of this approach vary greatly across cell lines and, for example, in human pluripotent stem cells (hPSCs), lentiviral or transposon‐mediated integrations are considerably more efficient [6]. However, one should consider that, to varying degrees, all three integration methods (i.e., spontaneous random integration, transposon‐ and virus‐mediated) can lead to disruption of actively transcribed genes and suffer from position effects [7, 8, 9], meaning that their expression will be strongly influenced by the chromatin surrounding the integration site [10] (Fig. 1). Position effects and epigenetic silencing processes are especially relevant when the genetic manipulation is performed in a precursor cell line that will then be differentiated into a specific cell type. In fact, during the process of differentiation changes in chromatin organization and epigenetic marks [11] can lead to the progressive silencing of transgenes [12]. Such effects can only be partially mitigated by the introduction of insulator sequences flanking our expression cassette [13, 14]. For such applications, the use of an episomal system containing the EBV‐encoded nuclear antigen‐1 (EBNA1) and oriP elements [15], minicircles [16], or S/MAR vectors [17, 18] viral delivery with adeno‐associated virus (AAV) [19, 20] or Sendai virus [21, 22, 23] particles may be advantageous, as they remain extrachromosomal but allow for sustained and long‐term transgene expression (Fig. 1). It is important to point out that, because their stability and retention are often limited in time and species‐dependent, episomal plasmids (e.g., EBNA‐based, S/MAR and minicircles) are not commonly used in standard laboratory practice. However, recently developed S/MAR vectors [24, 25] with improved retention and more robust expression might become a useful addition to the array of tools available for gene manipulation, especially where integrating and viral approaches are under very strict health and safety regulations.

### Stable expression and delivery methods

Sustained and steady transgene expression allows one to capture phenotypic changes requiring several days to manifest, which are typically difficult to observe by transient expression approaches. For example, siRNA knock‐down with a very efficient construct usually leads to a ~ 70–90% target reduction after 24–48 h, and beyond this point, the effects of the knock‐down will gradually decrease due to progressive dilution and clearance of the siRNA [26]. In the case of a very stable protein with a half‐life in the order of days, we would expect to see a sizable effect on the target protein levels only after 2–3 days, around the time that the effects of RNAi begin to wane. Therefore, in order to achieve robust knock‐down and capture any downstream phenotypic effects deriving from the loss of the target protein, two consecutive transfections may be employed [27]. Alternatively, one might choose to use any of the aforementioned systems for the sustained transgene expression of shRNAs against the target of interest. This is not the case in postmitotic cells, such as neurons, where plasmids and RNAs are not progressively diluted out through successive rounds of cell division, and transfection of standard plasmids yields long‐lasting transgene expression [28]. In this case, the main limitation is that the transfection efficiency of neurons, and generally primary cells, is very low, thus limiting the range of downstream analyses at one's disposal. In addition, transfection can be quite toxic to cells [28]. Alternative approaches are nucleofection and electroporation, which can be used to increase the delivery efficiency of naked DNA constructs, RNA, and ribonucleoprotein complexes (RNPs) [28, 29]. A clear advantage of electroporation over liposome transfection (i.e., lipofection) is the lower toxicity and the fact that it can be employed for delivery into tissues [30]: both *in vitro* differentiated from PSCs [22, 23], in acute preparations and slice cultures [31], or directly *in vivo*, to organs such as the brain [32], the vasculature, and lungs [33]. Nevertheless, using electroporation the delivery efficiency is usually not sufficient to perform bulk‐type analyses, such as biochemistry, proteomics, and genomics.

Transduction with AAVs or lentiviruses can be employed to achieve more efficient delivery of constructs and perform bulk‐type analyses. An additional advantage of using viruses, and specifically AAVs, is that the capsid type is an important determinant of tissue tropism [34]. Recently, using a multiplexed Cre‐recombination‐based AAV targeted evolution (M‐CREATE) platform, researchers were able to generate a range of capsid types that enable exquisite cell‐type‐specific expression of GFP under the control of a constitutive promoter [35]. The ability to encode such a high degree of cell‐type selectivity in the AAV capsid represents a substantial advance in the field of gene delivery and transgenesis and might aid in reducing the size of the promoter element required for expression in specific cell types. Nevertheless, the relatively small packaging limit (~ 4 kb) remains one of the main limitations of AAVs. For the delivery of larger inserts (i.e., up to ~ 10 kb), lentiviruses can be employed; although, they do not have serotypes with different tissue tropisms [36] (Fig. 1).

## Genome editing—beyond transgenesis

### The limitations of overexpression and knock‐down approaches

The approaches discussed so far are ideal for gain‐ and loss‐of‐function experiments where one can either overexpress or knock‐down a target gene via delivery of overexpression or RNAi constructs. Albeit very powerful for probing gene function, these approaches are relatively coarse, in that, ultimately, they only allow one to increase or reduce the levels of a given target and can produce nonspecific effects. For instance, RNAi can have off‐target effects due to nonspecific antisense binding to other cellular RNAs, and in some cases, RNAi has been reported to activate an interferon response [37] and alter endogenous miRNA pathways [38]. Therefore, the use of a complete set of controls, which includes nontargeting constructs and a non‐RNAi transfection control, is essential to discern genuine phenotypes. Albeit more challenging in terms of delivery, an alternative to RNAi is the use of morpholino oligos, which have higher specificity and do not engage the endogenous RNAi machinery but act by blocking the translation of the target RNA [39]. Beyond nonspecific effects, it is important to note that transient manipulations of target genes can lead to expression levels well outside a physiological range, which in itself might lead to artefactual phenotypes. For example, in the case where one may be interested in the function of a specific domain or set of amino acids in a protein, or a nucleotide sequence in a regulatory RNA species. The simplest and most common approach to tackle these questions is to overexpress a mutant construct in a wild‐type background and attempt to override the system to see the effects produced by the mutant protein or RNA species. However, by doing this, we are not simply changing the species present, we are also grossly altering overall target protein/RNA levels and this might give rise to artefactual dominant‐negative effects. A more informative approach is to replace the species in question with its alternative form; one way of doing so is to knock‐down or ‐out the endogenous copies of the gene‐of‐interest and then introduce a transgene encoding the mutant variant [40]. However, by this strategy the transgene introduced will not be under the control of the endogenous regulatory elements and its net expression dynamics might differ considerably from those of the endogenous gene.

### A direct handle on gene expression

A more elegant and powerful way of performing this type of replacement experiment, but also query the function of specific *cis‐*regulatory elements [41] and introduce expression cassettes at precise sites within the genome [42], is to use CRISPR‐Cas genome editing. This is a two‐component system comprising a CRISPR‐associated nuclease (Cas) and a guide RNA sequence (sgRNA), which targets the nuclease to a specific site in the genome [43, 44, 45]. After cleavage, the cells will try to repair the double‐strand break via different endogenous repair pathways and these can be exploited to introduce different types of mutations. Generally, gene knock‐out is achieved by the introduction of out‐of‐frame indels into the open reading frame (ORF) or excision of a critical exon by the nonhomologous end joining (NHEJ) repair pathway, while knock‐in is achieved by hijacking the endogenous homology‐directed repair (HDR) pathway to insert the desired repair template. The advantage of CRISPR‐Cas9 over zinc‐finger nucleases and TALENs [46] is its ease of programming. In fact, the sgRNA is a short synthetic RNA species comprising a scaffold, necessary for Cas binding, and a ~ 20 bp spacer sequence that specifies the genomic target site. For a sequence to act as a spacer it needs to be unique within the organism's genome and be directly adjacent to a protospacer adjacent motif (PAM)—in the case of Cas9 this is 5′‐NGG‐3′ [43, 44], while for Cas12a/Cpf1, a different CRISPR‐associated nuclease, the PAM site is TTTA/C/G [47]. There is a wealth of resources for sgRNA design that provide both an on‐target score, indicative of the efficiency of target site cleavage, and off‐target score, which reports on the likelihood of hitting unrelated genomic loci [48]. One way to limit potential off‐target effects is to use a mutated version of Cas9, Cas9 nickase (Cas9n), which can only introduce a single‐strand break or nick within the genome [49]. The rationale is that while off‐target nicks are efficiently repaired in mammalian cells, two sgRNAs targeting Cas9n to two genomic sites in close proximity will produce a double‐strand break (DSB) that can then be repaired by the cellular NHEJ or HDR pathways [49]. The increased specificity of nickase has been confirmed experimentally [50]; however, their main limitation is that activity can be strongly reduced compared with wild‐type Cas9 [50]. One aspect to consider is that inefficient editing will produce only few rare clones within a population of cells and their isolation, irrespective of any selection one might use, will inevitably require extended culture and sub‐pooling or single‐cell‐cloning steps. Because cells in culture spontaneously accumulate mutations [51, 52, 53], this might lead to genetic drift and the emergence of background mutations producing confounding artefactual phenotypes. In general, it is advisable to follow the simplest and most efficient editing strategy and to implement appropriate controls to identify genuine phenotypes. For instance, if one is interested in modeling the effects of complete or partial loss of a given gene, one valid strategy would be to use wild‐type Cas9 and, separately, three different sgRNAs to introduce indels within the target ORF by NHEJ. Following accurate genotyping, karyotyping, and assessment of target mRNA and protein levels, if the clones generated with the three distinct sgRNA display a similar phenotype, then this would increase the confidence that the gene‐of‐interest had been effectively targeted. In addition, a rescue experiment in which the target CDS is reintroduced by means of plasmid transfection or transduction [54, 55] would add further support to the findings. Rescue experiments can also help to rule out any effects arising from the disruption of enhancer elements at the target locus or from the emergence of a cryptic genetic variation. In addition to nickases, one way to reduce off‐target effects while maintaining high on‐target activity is to use DD‐Cas9, which enables conditional expression and temporal control of Cas9 levels by destabilization of the protein by FKBP12 synthetic ligands [56]. Another way to increase editing specificity is to use precomplexed Cas9 WT ribonucleoprotein complexes (RNPs) [57, 58]. In fact, while plasmid delivery is slow‐acting (i.e., it requires between 24 and 48 h to produce active nuclease complex) and long‐lasting (i.e., expression can last for up to a week), RNPs are active immediately after delivery and are cleared within 24 h [58]. By reducing the window of time cells express Cas9 both approaches reduce the frequency of off‐target effects (Fig. 1).

### dCas9 for transcriptional and epigenomic modulation

The epigenome, the collection of all epigenetic marks in the genome, together with transcription factors, determines the gene expression pattern of individual cells. Early attempts to modify the epigenome involved knock‐down/out and pharmacological inhibition of epigenetic modifiers [59]. These global approaches suffer from pleiotropic effects that do not allow targeted interrogation of the epigenetic state of a specific genomic region. Targeted transcriptional and epigenetic modulation became possible with the development of zinc‐finger proteins [60], TALENs [61], and nuclease‐deactivated Cas9 (dCas9) [62]. dCas9 in particular provides a flexible platform for site‐specific targeting by different modulation modalities including transcriptional blockade [63], gene expression modulation [64], epigenetic editing [65], and labeling of chromosomes for imaging [66]. In its simplest form, dCas9 can be used to interfere with RNA polymerase by steric blockade of transcription initiation or elongation [63, 67]. While this strategy is very effective in prokaryotes, in mammals it produces only modest effects [63]. To increase the potency of modulation in mammals, dCas9 can be fused to either a transcription activator domain (VP64, p65) for transcriptional activation (CRISPRa) or a repressor domain (KRAB, SID) for transcriptional silencing (CRISPRi) [62, 64], and multiple units of each are targeted to nearby genomic sites by using multiple sgRNAs. Modulation can be further enhanced by providing dCas9 with additional motifs, such as the SunTag [68] or RNA aptamers fused to the sgRNAs [69], so as to recruit a higher number of effector domain copies. Transcription repressors and activators modulate the target gene transcription indirectly by recruiting a host of epigenetic modifiers, chromatin remodelers, and secondary transcription factors. By contrast, epigenetic effectors (e.g., the DNA methyltransferase DMT3A, the histone demethylase LSD1, and the histone acetyltransferase p300 and CBP) fused to dCas9 alter the epigenetic state of the target in a direct and more predictable manner [62], without recruiting additional effectors. These approaches are extremely useful when one wants to probe how different epigenetic states affect gene expression, which is highly relevant in the context of development and disease. At the same time, by virtue of their rapid onset, robust effects and reversibility, CRISPRi, and CRISPRa represent alternatives to knock‐out/down and overexpression approaches to interrogate gene function.

### CRISPR for knock‐ins

In addition to loss‐of‐function experiments and epigenetic modulation, CRISPR‐Cas can be used in combination with homology‐directed repair (HDR) to perform scarless genome editing and insert new elements at specific genomic sites [43, 70]. While small (~ 50–200 bp) modifications only necessitate symmetric/asymmetric single‐stranded oligonucleotide donors (ssODNs) with 30–80 bp of homology, larger modifications (> 200 bp) are typically introduced by circular or linearized plasmids containing 500–1000 bp homology arms [43, 71] or with large ssODN derived by *in vitro* transcription and retrotranscription [72]. The decrease in the costs of gene synthesis and the establishment of ligation‐independent cloning methods, such as Gibson assembly [73] and In‐Fusion [74, 75] cloning, have considerably simplified the generation of large and complex templates for HDR. Importantly, the PAM site or the sgRNA seed sequence in the HDR template should be mutated so as to prevent nuclease cleavage. Additionally, in order to promote HDR over the more common NHEJ repair pathway, pharmacological treatment or a mutant version of Cas9 can be used [76]. An alternative repair pathway to HDR is microhomology‐directed end joining (MMEJ) [77], which is active during the M1 and S phases of the cell cycle, and is error‐prone but has the advantage of requiring only 5–30 bp of homology. Due to their cell‐cycle phase requirements, HDR and MMEJ have limited suitability for genome editing in postmitotic cells such as neurons [76]. HDR has been used successfully to fluorescently tag synaptic proteins in neurons, both at the neural progenitor state [78] and after cell‐cycle exit [79]. However, it should be noted that the HDR pathway is scarcely active in postmitotic cells and successful knock‐in requires neurons derived from Cas9 knock‐in mice and AAV‐mediated delivery of the HDR template. To provide some context, it was estimated that postmitotic neurons require a ~ 100‐fold higher concentration of repair template compared with neural progenitors [79]. For targeted genomic integration directly in neurons, the alternative NHEJ‐based homology‐independent targeting integration (HITI) [80] repair approach was shown to be more efficient (Fig. 1). Unlike HDR, HITI does not yield predictable repairs, as small indels are introduced along with the insert [81]. Systems such as HiUGE [80], ORANGE [82], and CAKE [83] are based on HITI and enable tagging and manipulation of endogenous synaptic proteins in neurons. Editing strategies that can overcome potential problems with out‐of‐frame repair are TKIT [84] and CRISPIE [85], which target intronic regions and thus are less sensitive to frame changes. Whilst these approaches are powerful for microscopy studies, delivery issues combined with the low efficiency of correct repair leading to tagging, complicate the use of such strategies for bulk‐type approaches. Furthermore, it remains difficult, if not impossible, to distinguish between monoallelic vs. biallelic targeting, which complicates the interpretation of the results. All these limitations stem from the fact that postmitotic cells cannot be single‐cell cloned, expanded, and genotyped.

## Transgenic animals and *in vitro* differentiation

As previously mentioned, immortalized cell lines are a valuable tool for studies of gene function; however, due to the high rates of aneuploidy and spontaneous mutations, observations made using such models should be ultimately tested in systems that more closely capture normal cell physiology. In this regard, the generation of transgenic animals [86] allows for precise genomic manipulations in a more physiologically relevant context. The use of conditional knock‐outs by tissue‐specific recombination [87], engineering of inducible expression cassettes at safe‐harbor loci (i.e., intra‐ and intergenic genomic regions that enable the stable expression of integrated transgenes) [88], precise excision of specific enhancer regions from the germline [41] and the establishment of complex genetic reporter systems for lineage tracing studies [89, 90] are some of the examples that showcase the power of mouse models. Nevertheless, generating a mouse model can be a considerable financial undertaking, it is particularly cumbersome and time‐consuming and ultimately it may not yield the expected results (Fig. 1). An attractive alternative to bridge the gap between standard *in vitro* preparations and *in vivo* models are 2D [91, 92, 93] and 3D [94, 95, 96, 97, 98] *in vitro* differentiated PSC‐derived cultures. These models capture more closely the complex cellular interactions found in tissues [98, 99, 100] and allow the study of both transient and mature cell types. In addition to modeling both normal physiology and pathology with higher fidelity than immortalized cell lines, such differentiation approaches can be easily implemented in a standard laboratory setting in a cost‐effective manner. The fact that PSCs can be derived from several different sources with minimally invasive procedures has led to the establishment of PSC lines from many different species, thus allowing cross‐comparative and functional evo‐devo studies [54, 101, 102, 103]. An important advance for such studies has been the recent development of PSC‐culture media suitable for the culture of cells from different mammalian species [54] and that can significantly improve cell viability under conditions of stress [104, 105], such as those encountered during single‐cell‐cloning procedures for genome editing applications. Although editing human PSCs remains challenging, efficient delivery of WT Cas9 plasmid constructs and RNPs by electroporation or lipofection followed by clonal selection with StemFlex supplemented with RevitaCell [106] on plates coated with rhLaminin‐521 [104, 107] is generally sufficient to isolate NHEJ knock‐out clones from one to two 96 well plates without any enrichment strategy or selection. For more complicated applications involving HDR or MMEJ, antibiotic selection or FACS can greatly expedite the isolation of mutants. However, in many cases, the target gene is not expressed in PSCs and thus fluorescent tagging or fusion to an antibiotic resistance cassette via a self‐cleaving tag are not viable options for enrichment. Another instance in which selection is not possible is in the case of scarless genome editing to generate cells that differ only at the engineered mutation site. In such cases, TaqMan probe‐based assays by ddPCR and sib selection (i.e., a method to isolate clones from a population based on the repeated fractionation and selection of a positive subfraction) [108] can be used to progressively enrich and purify edited clones (Miyaoka *et al*. [109]). However, it should be noted that these approaches are laborious and time‐consuming, so wherever possible a more ‘quick and dirty’ approach is advisable (Fig. 1).

## Targeted protein degradation (TPD) for rapid and acute protein removal

So far, all the perturbation strategies discussed act at the level of genes and their transcripts. They provide a means to directly manipulate the target when one wants to modify a certain genomic region or RNA species. However, in the case of protein‐coding genes, they affect changes in protein levels indirectly through their DNA or RNA precursor. As a consequence, the efficacy of the modification will depend on the transcript and protein half‐life; compensatory feedback mechanisms might be invoked and, invariably, there will be a lag between the time of perturbation and the time phenotypic changes start to manifest [110]. Such shortcomings are particularly restrictive when we want to explore the consequences of acute protein depletion, which is highly relevant in the case of dynamic and transient processes such as those seen during development and in response to cellular stimuli and insults. Advances in chemical biology have made it possible to use the proteasomal and endo‐lysosomal protein degradation pathways to rapidly destabilize soluble intra‐ and extracellular proteins, as well as membrane proteins with dose tunability, reversibility, and high selectivity (Fig. 1) [110, 111, 112, 113]. Since the array of systems and technologies developed is vast and ever‐expanding, we provide a general conceptual framework and highlight systems that can be easily implemented in a laboratory setting, using off‐the‐shelf reagents, without the need for screening protein binders and synthesizing novel compounds. The idea underlying all TPD approaches is the use of a bifunctional compound or antibodies to bring the protein of interest (POI) into close proximity with an effector that triggers its degradation by either the ubiquitin‐proteasome system (UPS) or the endo‐lysosomal pathway [111, 112]. Approaches for the destabilization of intracellular proteins include proteolysis targeting chimeras (PROTACs), autophagy‐targeting chimeras (AUTACs), and autophagosome tethering compounds (ATTECs; Fig. 1). PROTACs [114] and AUTACs [115] consist of a chemical warhead, which binds the POI, fused via a flexible chemical linker to an E3 ligase ligand or a guanine derivative, respectively. ATTECs [116, 117] are linker compounds that tether the POI to LC3 proteins of the phagophore, during autophagosome formation. Destabilization of extracellular and membrane proteins can be achieved with lysosomal targeting chimeras (LYTACs) or antibody‐based PROTACs (AbTACs) (Fig. 1). While LYTACs [118] comprise a chemical warhead that binds the POI fused to a lysosome targeting receptor ligand, AbTACs [119] are recombinant bispecific antibodies that bind both the POI and the transmembrane E3 ligase RNF43 (Fig. 1). In contrast to PROTACs, which recruit E3 ubiquitin ligases that catalyze K48 polyubiquitination of the POI and engage the UPS, the mechanism of action of AbTACs remains elusive and it is not clear whether RNF43 triggers ubiquitination of the POI to initiate internalization [112]. The development of such tools is a considerable chemical biology undertaking in that it requires the identification of a specific ligand of the POI, optimization of the linker, and identification of an effector able to degrade the POI; all this while maintaining good bioavailability [110]. The investment necessary to develop these reagents is such that at present available TPD tools target only a relatively small set of POIs, many of which are disease‐relevant and of interest to the pharma industry. For discovery biology, an approach to bypass these hurdles is to employ genome editing to fuse the endogenous gene‐of‐interest with a HaloTag [120], a degradation tag (dTAG) [121, 122], an Achilles TAG (aTAG) [123] or the auxin‐inducible degron (AID) [124, 125], among others. These tags endow the POI with ‘standardized’ domains that can be bound by predesigned and commercially available degrader molecules, which recruit different E3 ubiquitin ligases (i.e., VHL, CRBN, OsTIR1(F74G)) to initiate degradation by the UPS (Fig. 1). Albeit conceptually similar, an important difference between the AID and the other TAG systems is that while the former is a plant‐derived system [124, 125] that requires an exogenous E3 ubiquitin ligase (TIR1 or OsTIR1(F74G)), the latter systems hijack the endogenous and ubiquitously expressed E3 ligases VHL or CRBN. These tags are relatively small and they should not impact POI function. The suitability of a given system for POI destabilization will depend on the cell type expression, intracellular localization, and biochemical properties of the POI and E3 ligase pair. Preliminary overexpression of the POI‐TAG fusion in the cell type of choice is essential to identify the optimal destabilization system before performing genome engineering [122]. While UPS‐recruiting TAG systems are now well supported by a range of off‐the‐shelf reagents, this is not the case for TPD systems that rely on the endo‐lysosomal protein degradation pathway. However, spurred by its therapeutic potential, the field of TPD is rapidly growing and we anticipate that more plug‐and‐play systems based on a variety of degradation modalities will make their way into discovery biology in the near future [110]. Lastly, valuable TPD strategies that do not rely on chemical degraders and do not require cumbersome genome editing are antibody‐based approaches such as Trim‐Away [126] and FingR intrabodies‐E3 ligase fusions [127]. Being entirely genetically encodable, the latter tool is particularly powerful as it can be packaged into viruses for efficient delivery and combined with a Tet‐On inducible promoter for tunability.

## Conclusions and outlook

In this short piece, we have discussed some of the technologies and methodologies available to explore target gene function across a range of different mammalian systems. Due to space constrains, we only focused on those that we routinely use in our line of research, but we hope that the breadth of topics covered will serve as a starting point and orient the reader on how to best tackle their scientific question. It should be noted that all approaches and systems come with advantages and disadvantages and these should be weighed carefully. Generally, a sensible strategy is to start from the simplest system in which a given process can be modeled to then expand to more complex and cumbersome ones to address very targeted questions that could not be answered otherwise (Fig. 1). With the discovery of RNAi, CRISPR‐Cas and TPD technologies the past 20 years have witnessed a revolution in the way we can dissect signaling pathways. Now, the development of base editors [128], CRISPR enzymes that enable manipulation of RNA [129, 130, 131], and many other variations [132] promise to take this to the next level. At the same time, the rise of *in vitro* PSC‐differentiation models has provided an ideal platform to make the most of such approaches [98]. At present, the use of these models cannot replace animal studies, and it is difficult to envisage a future where they will entirely; however, their value is unquestionable and we anticipate that they will increasingly become a powerful complement to animal studies in both basic and translational research.

## Conflict of interest

The authors declare no conflict of interest.

## Author contributions

SLG and EMS wrote the review, and SLG designed the figures.

## Data Availability

None.
